# Disease Burden of 30 Cancer Groups in Taiwan from 2000 to 2021

**DOI:** 10.1007/s44197-025-00406-w

**Published:** 2025-04-22

**Authors:** Po-Chen Liu, Tzu-Hsuan Huang, Yun-Chun Wu, Yueh Wang, Chun-Ju Chiang, Wen-Chung Lee, Hsien-Ho Lin, Wei-Cheng Lo

**Affiliations:** 1 Institute of Epidemiology and Preventive Medicine, College of Public Health, National Taiwan University, Taipei, Taiwan; 2 Master Program in Applied Epidemiology, College of Public Health, Taipei Medical University, Taipei, Taiwan; 3 Population Neuroscience and Genetics Lab, Center for Human Development, UC San Diego, San Diego, CA USA; 4 Taiwan Cancer Registry, Taipei, Taiwan; 5 Master of Global Health Program, College of Public Health, National Taiwan University, Taipei, Taiwan; 6 School of Public Health, College of Public Health, Taipei Medical University, Taipei, Taiwan

**Keywords:** Burden of cancer, Disability-adjusted life years, Life expectancy decomposition

## Abstract

**Background:**

Assessment of the morbidity and mortality burden of cancers and their evolving trends is crucial for making informed policy decisions and effective resource allocation. We aimed to examine the burden of cancer in Taiwan from 2000 to 2021 using a national population-based database.

**Methods:**

Linking data from the Taiwan Cancer Registry and National Death Registry, we calculated years lived with disability (YLDs), years of life lost (YLLs), and disability-adjusted life years (DALYs) for 30 specific cancer groups. Our methodology aligns with the Global Burden of Disease Study.

**Results:**

In Taiwan, from 2000 to 2021, the age-standardized cancer mortality rate decreased by 13.8%, while the prevalence rate increased by 80.5%. In 2021, the age-standardized DALYs for total cancer were 3784.2 per 100,000 population. In 2021, in Taiwan, lung, liver, and colorectal cancers were the leading contributors to cancer-related DALYs for men, whereas breast, lung, and colorectal cancers were predominant for women. Life expectancy decomposition analysis revealed distinct patterns by sex, with significant gains for specific cancers from 2000 to 2021: cervical, stomach, and liver cancers in women (0.20, 0.13, and 0.12 years, respectively) and liver, lung, and stomach cancers in men (0.37, 0.17, and 0.17 years, respectively).

**Conclusion:**

Our finding of declining cancer DALY rates in Taiwan over the past two decades may reflect improvements in cancer control, particularly the significant decrease in liver and lung cancer burden. However, the rising burden of breast cancer and the sustained impact of colorectal and oral cancers warrant targeted attention in health policies, resource allocation, and research to reduce healthcare costs and improve quality of life.

**Supplementary Information:**

The online version contains supplementary material available at 10.1007/s44197-025-00406-w.

## Introduction

Cancer is one of the major causes of death worldwide. In 2021, cancer caused approximately 9.9 million deaths and resulted in 253.3 million disability-adjusted life years (DALYs) worldwide [1]. According to the annual report from the Taiwan Cancer Registry, 121,762 new cancer cases and 51,656 cancer-related deaths occurred in Taiwan in 2021 [2]. Over the past few decades, the implementation of various cancer control strategies in Taiwan, including primary prevention [15], early detection and screening [17], and improvements in the quality of diagnosis and treatment services, has substantially reduced the overall cancer mortality rate [2, 3, 16, 18, 19]. With an increase in the survival rate of patients with malignant neoplasms, particularly those detected through screening, including colorectal, female breast, cervical, and uterine cancer, increasing attention has been focused on improving patients’ quality of life by reducing cancer-related sequelae and disability. Moreover, improvements in life expectancy and an aging population are expected to increase the cancer burden in Taiwan. Thus, the burden and trends of cancer should be systematically evaluated to identify gaps and challenges and determine the effectiveness of interventions. However, comparative evidence on cancer burden obtained using various health metrics remains limited. In addition, most studies on cancer epidemiology conducted in Taiwan have focused only on a single or few cancer sites or on incidence and survival patterns.

DALYs as a measure of disease burden, are composed of years lived with disability (YLDs) and years of life lost (YLLs), providing a quick, ballpark view of the burden of cancer with information derived from fatal and non-fatal outcomes. The Global Burden of Disease (GBD) study has reported incidence rates, mortality rates, YLLs, YLDs, and DALYs for 34 cancer groups in 204 countries [1, 4]. Although the GBD study provided a consistent framework to assess and compare disease burdens across countries, the accuracy of country-level estimates may be compromised due to limited data accessibility. Thus, to address data gaps, the GBD study used a standardized estimation approach involving spatial–temporal disease modeling with Bayesian meta-regression [1, 4]. Although this approach produces consistent and comparable global estimates, country-specific estimates can be considerably biased if the underlying assumptions of the model are violated. Hence, to obtain more plausible country-level estimates, many countries, such as Australia, New Zealand, China, South Korea, Japan, and Singapore, have conducted their own national or subnational burden of disease studies by using local health data unavailable to the GBD study team [5–10]. Most of these studies have followed the GBD study protocol with minor adjustments to better fit the local context and obtain accurate country-level estimates while maintaining their comparability at the global level.

In the present study, we obtained our cohorts by linking data from the Taiwan Cancer Registry and National Death Registry. We clearly defined the status and corresponding disability weight (DW) for each patient with cancer. We estimated DALYs, which represent both morbidity and mortality burdens for all cancer sites, individually and collectively. In addition, we conducted time trend analyses for DALYs and other health metrics to examine changes in cancer burden over time.

## Methods

### Data Sources and Study Cohort

We estimated the burden of 30 types of cancer in Taiwan from 2000 to 2021 by using data from the Taiwan Cancer Registry and National Death Registry. In Taiwan, all deaths are registered in accordance with laws and regulations, and information on the individual causes of death was obtained from the Department of Statistics, Ministry of Health and Welfare. The Taiwan Cancer Registry, a population-based registry, covers almost all hospitals with a capacity of more than 50 beds, ensuring nationally representative data. Since 2012, data completeness has exceeded 98%, and since 2008, the morphological verification rate (MV%) has remained above 90%. Additionally, since 2011, the DCO% (the proportion of case with only death certificate) has been maintained below 0.8% [11]. We used the cause of death dataset to estimate the fatal disease burden, measured in YLLs. In addition, by combining data on newly diagnosed cancer cases from the Taiwan Cancer Registry with individual cause of death data, we applied the disease model from the Australian Burden of Disease Study [6] to estimate the nonfatal disease burden, measured in YLDs.

### Estimation of Fatal Disease Burden

Before 2008, the causes of death were coded in accordance with the *International Statistical Classification of Diseases and Related Health Problems*,* 9th Revision*, which recorded a single underlying cause of death. However, from 2008 onward, multiple causes of death began to be recorded in accordance with the *International Statistical Classification of Diseases and Related Health Problems*,* 10th Revision*. To manage this transition, we aligned the cause of death with our previously established mapping lists, categorizing them accordingly. Following the methodology employed in our previous study, we used a naïve Bayes classifier in a garbage code redistribution model to refine the data [12]. Figure S1 presents a comparison of the mortality rate by cancer type before versus after garbage code redistribution. After redistributing the causes of death, we grouped the data into 5-year age groups. These grouped data were then multiplied by life expectancy estimates obtained from the standard life table of the GBD study to calculate YLLs.

### Estimation of Nonfatal Disease Burden

Disease classification was performed in accordance with the *International Classification of Diseases for Oncology*,* Third Edition* (Table S1) by using data from the Taiwan Cancer Registry. Benign neoplasms were excluded on the basis of the fifth character of the morphology code. Patients included in the YLD model underwent a sequential series of phases in the disease process (Figure S2). (1) Diagnosis and Treatment Phase ($\:{T}_{DT}$): This initial phase begins at disease onset, when patients receive their diagnosis and start receiving treatment. (2) Remission Phase ($\:{T}_{R}$): After the diagnosis and treatment phase, patients enter the remission phase. (3) Hypothetical Cure Phase ($\:{T}_{C}$): If a patient survives for 10 years after diagnosis, they transition into this phase. (4) Metastatic ($\:{T}_{M}$) and Terminal Phases ($\:{T}_{T}$): If a patient’s cancer progresses, they move through the metastatic phase and eventually enter the terminal phase at the end of life. All cancers are assigned the same DW for each phase, but the duration of each phase varies by cancer type. In particular, only the terminal phase has a fixed duration, set at 1 month prior to death from cancer. The duration of the remission phase is calculated as the residual time remaining after subtracting the durations of the other three phases from the total time since diagnosis. The duration of the terminal phase is prioritized over the metastatic phase, which, in turn, takes precedence over the diagnosis and treatment phase.

The individual diagnosis date for each patient with cancer, as recorded in the Taiwan Cancer Registry database, served as the starting point for the diagnosis and treatment phase. The beginning of the terminal phase was determined on the basis of a patient’s date of death, which was obtained by linking to the National Death Registry dataset. The definitions of all phase durations and DWs for all phases were obtained from the 2021 GBD study (Tables S2 and S3). After establishing the disease process for each patient, we estimated the point prevalence of disease phases for different cancer groups on July 1^st^ of each year. Subsequently, we calculated the nonfatal disease burden by multiplying the phase-specific DW by the sum of the point prevalence for each phase.

### Effect of Cancer Mortality on the Increase in Life Expectancy

We estimated and decomposed life expectancy by using the method reported by Chiang [13] to determine the increase in life expectancy at birth by comparing cancer mortality in 2021 with that in 2000. We individually estimated the expected mortality for each cancer type in 2021 on the basis of the age-specific mortality rate in 2000 for that specific cancer type. We assumed no interactions among different cancer types during this analysis. To determine the influence of each cancer type on life expectancy, we calculated the contribution of each cancer type by dividing the difference in life expectancy between 2000 and 2021 by the life expectancy in the initial year, 2000. The resulting percentage indicated the proportion of improvement in life expectancy that could be attributed to changes in the mortality rate of a specific cancer.

### Reporting Standards

DALYs were calculated by combining YLLs and YLDs. We computed annual rates by dividing the absolute DALY estimates by the mid-year population. To enable comparisons both within and between countries, we used the age structure of the standard population provided by the Institute for Health Metrics and Evaluation [1]. In addition, we determined the average life lost by dividing the absolute YLLs by the total number of deaths, and this measure provided an estimate of the average amount of time lost per individual due to death from specific cancer types. The adjusted DW was calculated by dividing the absolute YLDs by the cancer prevalence, and this measure indicated the average number of years that a patient lost due to cancer-related disability. All analytical steps and estimates reported in this study adhered to the Guidelines for Accurate and Transparent Health Estimates Reporting (GATHER) [14].

## Results

### Prevalence, Mortality, and DALYs of Cancer Types

In 2021, the age-standardized prevalence, mortality, and DALYs for all cancer types were 2252.7, 156.4, and 3784.2 per 100,000 population, respectively (Table 1 and Figure S4–S8). In 2000, these estimates were 1247.7, 181.5, and 4555.7 per 100,000 population, respectively. In 2021, of the total DALYs attributed to cancer, 95.9% were from YLLs and 4.1% from YLDs. Table 1 presents the distributions of cancer burden across 30 cancer groups. Lung cancer had the highest contribution, accounting for 15.1% of total cancer burden, followed by breast (13.1%), liver (12.2%), and colorectal cancer (10.6%) in 2021. The primary contributors to cancer-related DALYs were lung, liver, and colorectal cancers for men and breast, lung, and colorectal cancers for women.

**Table 1:** Age-standardized rate per 100,000 population for mortality, prevalence, years of life lost, years lived with disability, and disability-adjusted life years in Taiwan, 2000 and 2021

	Age-standardized rate (per 100,000 population) for both sexes										
	Mortality		Years of life lost		Prevalence		Years lived with disability			Disability-adjusted life years	
Cancer groups/year	2000	2021	2000	2021	2000	2021	2000	2021	2000		2021
Tracheal, bronchus, and lung cancer	34.50	27.33	719.25	559.40	60.52	149.30	8.90	13.91	728.15		573.30
Breast cancer*	11.17	15.63	367.30	451.75	216.99	598.21	19.27	42.11	386.57		493.86
Liver cancer	31.17	21.06	812.19	453.29	76.40	110.37	8.90	9.34	821.10		462.63
Colon and rectum cancer	19.05	18.34	402.32	378.84	153.87	261.24	15.55	21.55	417.87		400.39
Lip and oral cavity cancer	5.69	6.75	185.37	197.94	48.80	106.18	5.66	9.40	191.04		207.34
Esophageal cancer	4.62	5.49	121.18	159.08	10.87	20.05	1.67	2.54	122.85		161.62
Prostate cancer*	7.74	10.39	114.69	136.63	87.87	252.93	12.10	21.57	126.79		158.19
Pancreatic cancer	5.40	7.14	117.39	156.04	6.79	13.33	1.09	1.90	118.48		157.94
Stomach cancer	13.40	6.22	275.36	127.05	61.72	44.40	6.16	3.96	281.53		131.01
Ovarian cancer*	3.27	3.68	94.64	107.27	32.17	62.11	3.63	5.65	98.27		112.92
Uterine cancer*	1.42	2.96	38.27	83.29	29.43	109.63	2.28	7.68	40.55		90.96
Other pharynx cancer	1.58	2.78	48.94	82.36	9.60	25.03	1.17	2.43	50.11		84.79
Non-Hodgkin lymphoma	4.11	3.62	114.86	79.10	14.41	41.87	1.33	3.32	116.19		82.42
Cervical cancer*	10.00	2.79	255.03	73.78	185.44	53.64	14.29	4.07	269.32		77.85
Brain and central nervous system cancer	2.30	1.94	87.19	74.95	11.75	13.45	1.14	1.32	88.33		76.27
Kidney cancer	2.26	2.41	51.70	54.96	28.86	49.97	2.43	3.67	54.14		58.62
Nasopharynx cancer	3.96	1.82	126.12	55.17	36.74	32.60	3.80	2.72	129.91		57.89
Bladder cancer	3.55	3.01	62.22	48.32	42.09	34.66	3.52	2.75	65.74		51.07
Acute myeloid leukemia	0.00	1.67	0.01	48.55	4.88	7.56	0.44	0.70	1.73		49.25
Gallbladder and biliary tract cancer	4.50	1.66	98.19	32.78	7.00	7.74	0.76	0.78	98.96		33.56
Other leukemia	4.10	0.94	147.56	23.24	0.40	0.03	0.04	0.00	143.53		23.24
Acute lymphoid leukemia	0.00	0.40	0.01	19.63	4.69	1.97	0.49	0.18	3.12		19.80
Thyroid cancer	0.68	0.59	13.52	11.17	33.72	111.39	2.24	6.33	15.77		17.50
Larynx cancer	1.18	0.69	27.89	15.33	12.08	11.86	1.11	0.92	29.00		16.25
Testicular cancer	0.06	0.19	2.83	9.77	5.24	20.39	0.37	1.25	3.20		11.02
Chronic myeloid leukemia	0.00	0.18	0.01	4.26	0.01	5.94	0.00	0.40	0.01		4.66
Mesothelioma	0.00	0.18	0.00	3.93	0.27	0.58	0.02	0.10	0.02		4.02
Chronic lymphoid leukemia	0.00	0.14	0.01	2.26	1.32	3.78	0.10	0.33	0.27		2.60
Hodgkin lymphoma	0.10	0.04	3.24	1.07	1.70	6.66	0.14	0.39	3.38		1.46
Others	5.66	6.31	144.86	154.93	62.07	95.79	4.93	6.84	149.79		161.76
Total	181.48	156.35	4,432.18	3,606.11	1,247.68	2,252.66	123.55	178.09	4,555.73		3,784.20

*Rates for sex-specific cancer types were calculated using only the female population for cervical, ovarian, uterine, and breast cancers and only the male population for prostate and testicular cancers

**Fig. 1:**
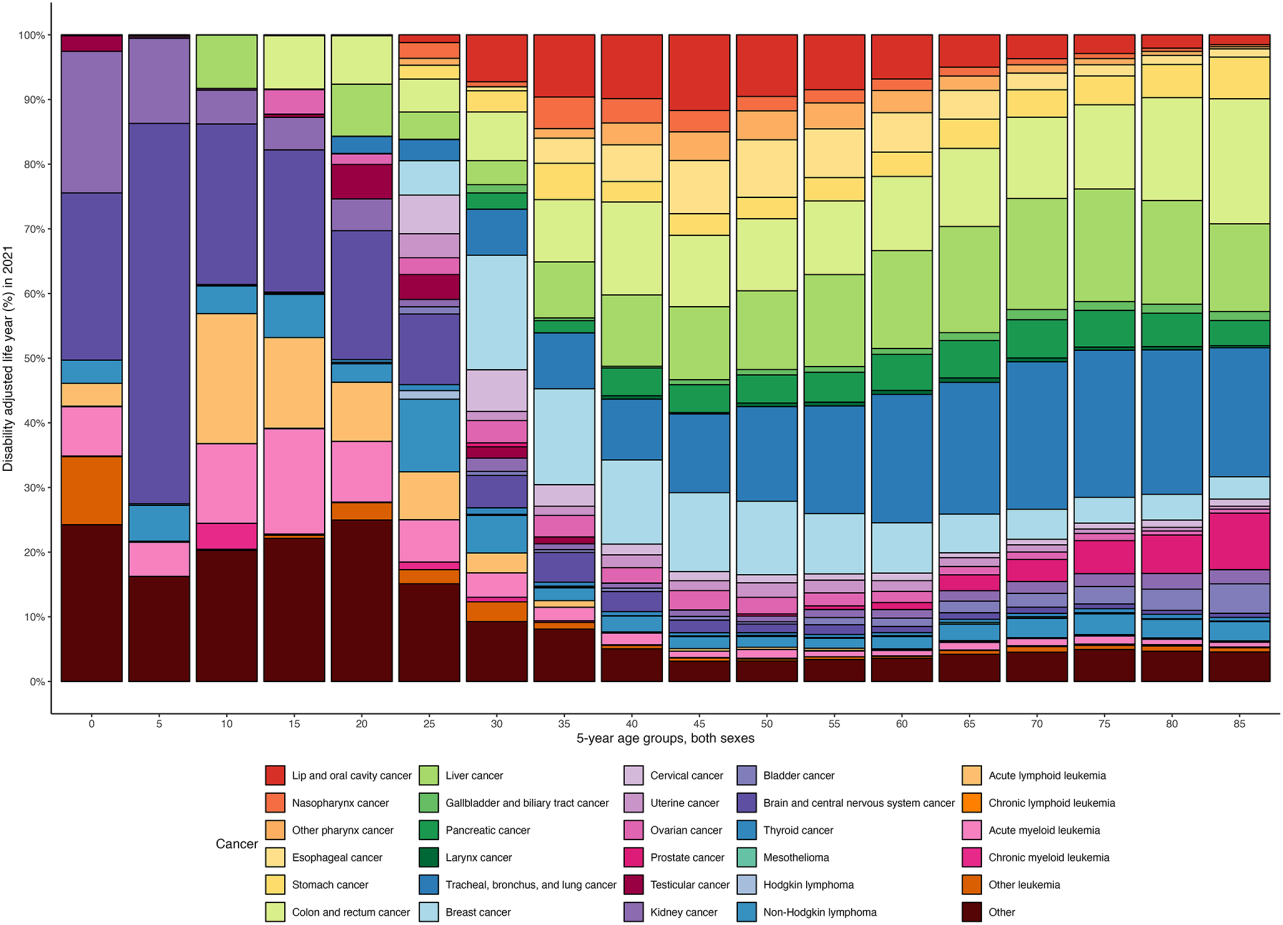
Age-specific contributions of different cancer types to total cancer-related DALYs for both sexes in 2021 in Taiwan

Figure 1 illustrates the contribution of various cancer types to total cancer burden by age group. In children and adolescent (aged 0–19 years), the main contributors to total cancer-related DALYs were kidney, brain and central nervous system cancers, and acute lymphoid leukemia. Among young and middle-aged adults (aged 20–59 years), the main contributors to cancer-related DALYs were lung, liver, colorectal, and breast cancers. Among those older than 59 years, the major drivers of DALYs were lung, liver, and colorectal cancers.

### Changes Over time

Different patterns in the leading cancer sites for age-standardized YLD, YLL, and DALY rates were observed across the study period (from 2000 to 2021). In 2021, the leading contributors to the YLD burden were breast, prostate, colorectal, lung, oral, and liver cancers, demonstrating percentage changes of 118.5%, 78.3%, 38.6%, 56.2%, 66.0%, and 4.9% from 2000 to 2021, respectively. (Figure S6) For age-standardized YLL rates over time, lung, breast, liver, and colorectal cancers remained the top four leading contributors in both 2000 and 2021. Oral cancer moved from the seventh to the fifth position, pancreatic cancer from the twelfth to the seventh position, and esophageal cancer from the eleventh to the sixth position (Figure S7). A consistent ranking pattern was observed for age-standardized DALY rates. We noted a 21.3% decrease in lung cancer DALYs, a 27.8% increase in breast cancer DALYSs, a 43.7% decrease in liver cancer DALYs, a 4.2% decrease in colorectal cancer DALYs, an 8.5% increase in oral cancer DALYs, and a 24.8% increase in prostate cancer DALYs from 2000 to 2021 (Figure S8).

### Spectrum of Morbidity and Mortality Burden Due To Cancer

Figure 2a displays a scatter plot of prevalence versus adjusted DWs for 30 cancer groups, with a color spectrum classifying different levels of morbidity burden. In this plot, contours were defined by the first and third quartile values of YLLs or YLDs, which were the products of the two axes. Cancers with YLL or YLD values above the first quartile were categorized as high burden diseases. Those with values between the first and third quartiles were categorized as medium burden diseases, and cancers below the third quartile were considered low burden diseases. In 2021, among the cancer groups with a high morbidity burden, breast cancer had the highest prevalence (0.598%), while lung cancer exhibited the most severe adjusted DW (0.093) in the high burden category. As presented in Fig. 2b, a scatter plot of mortality and average life lost for the 30 cancer groups revealed that lung, liver, colorectal, breast, oral, pancreatic, and esophageal cancers fell within the high mortality burden group. Lung, liver, and colorectal cancers had the highest mortality rates in the Taiwanese population in 2021. Furthermore, among those high cancer burden groups, oral cancer resulted in a greater average loss of life years (29.3 years) than did other cancer types.

**Fig. 2a:**
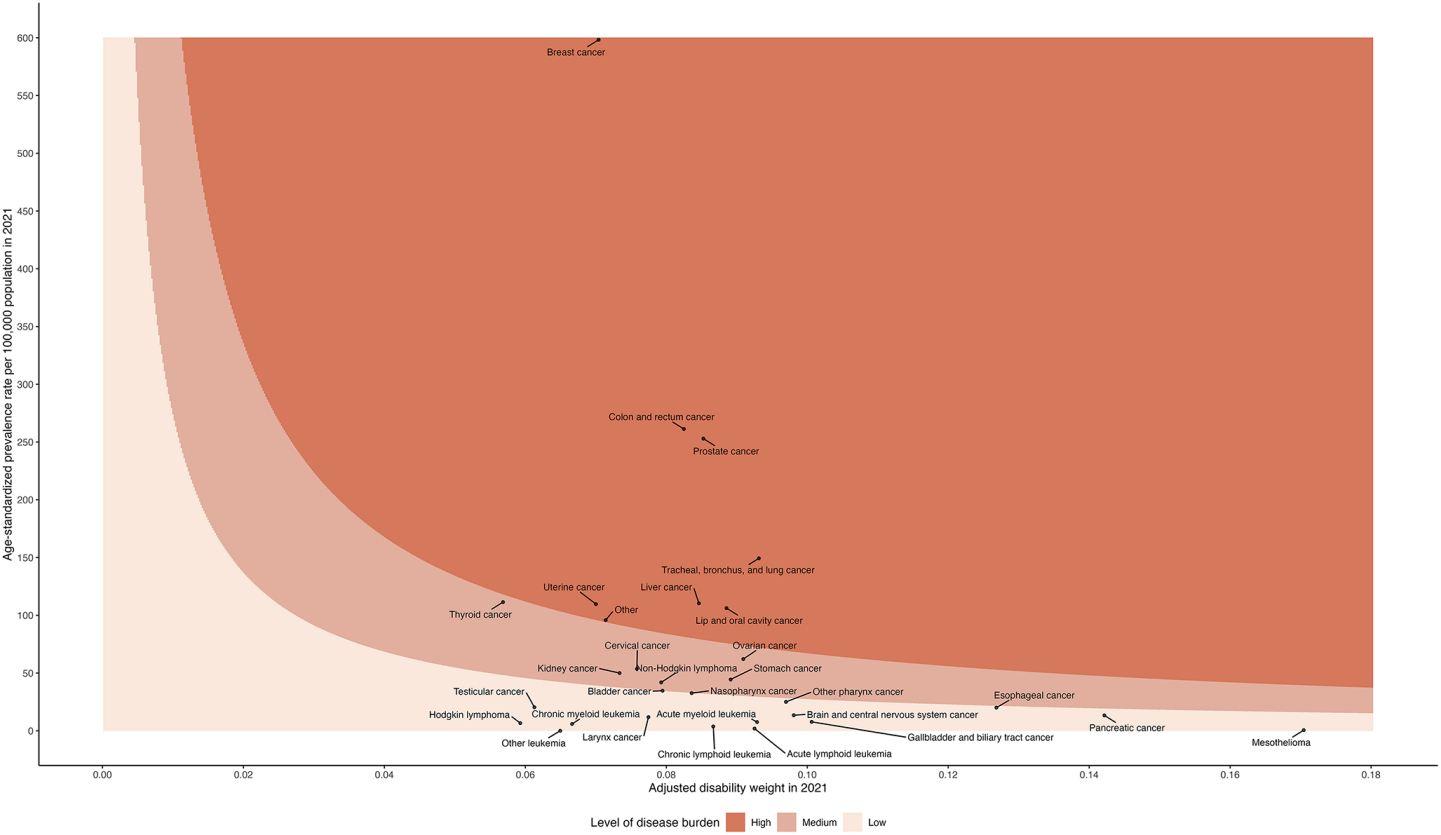
Spectrum of nonfatal cancer burden in 2021 for both sexes in Taiwan. Y-axis represents the age-standardized prevalence, and X-axis represents the severity-adjusted disability weights of each cancer type. The contours were defined by the first and third quartile values of YLDs, which were the products of the two axes

**Fig. 2b:**
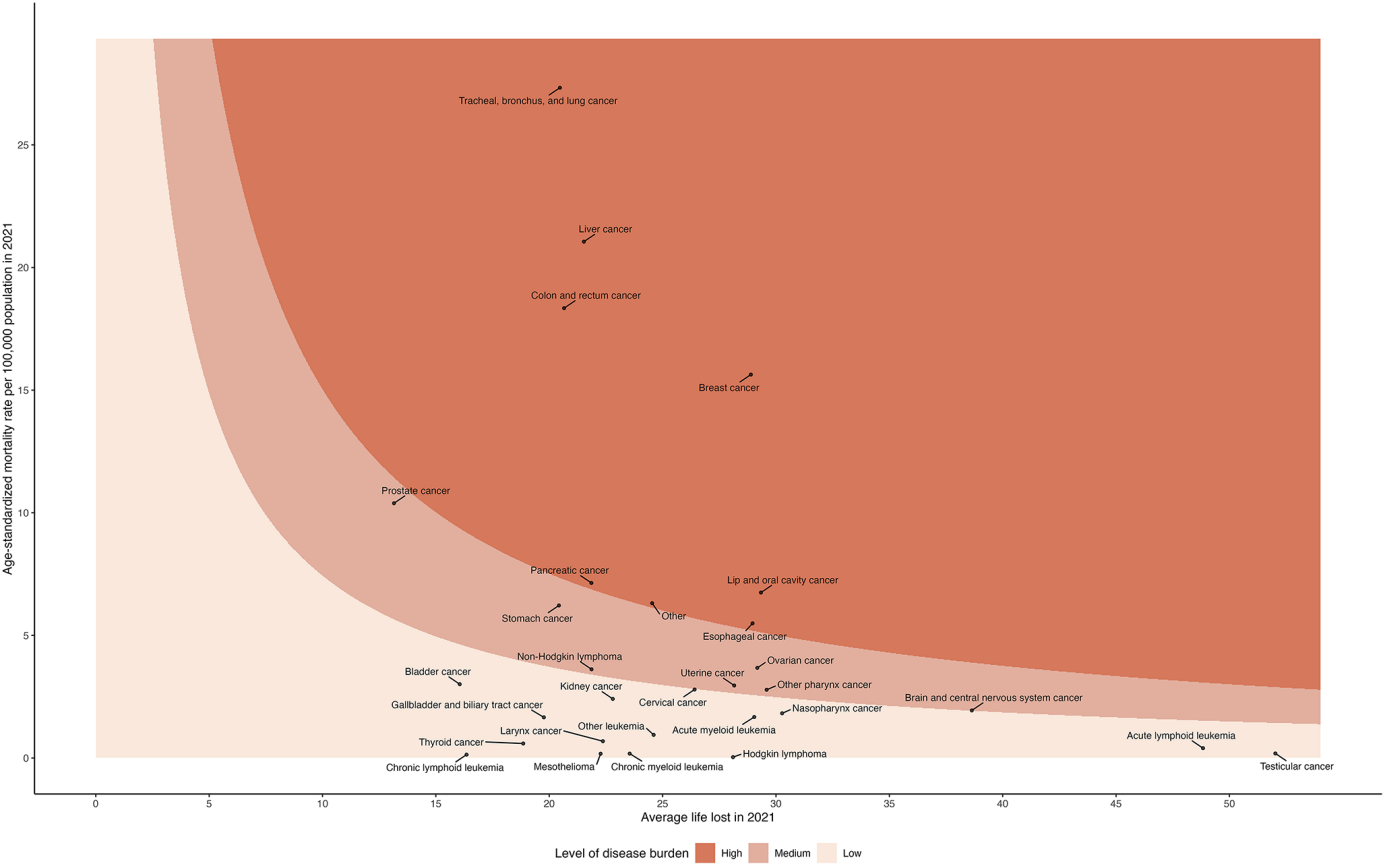
Spectrum of fatal cancer burden in 2021 for both sexes in Taiwan. Y-axis. represents the age-standardized mortality, and X-axis represents the average of years of life lost for each cancer type. The contours were defined by the first and third quartile values of YLLs, which were the products of the two axes

### Decomposition of Changes in Life Expectancy Due To Cancer

**Fig. 3:**
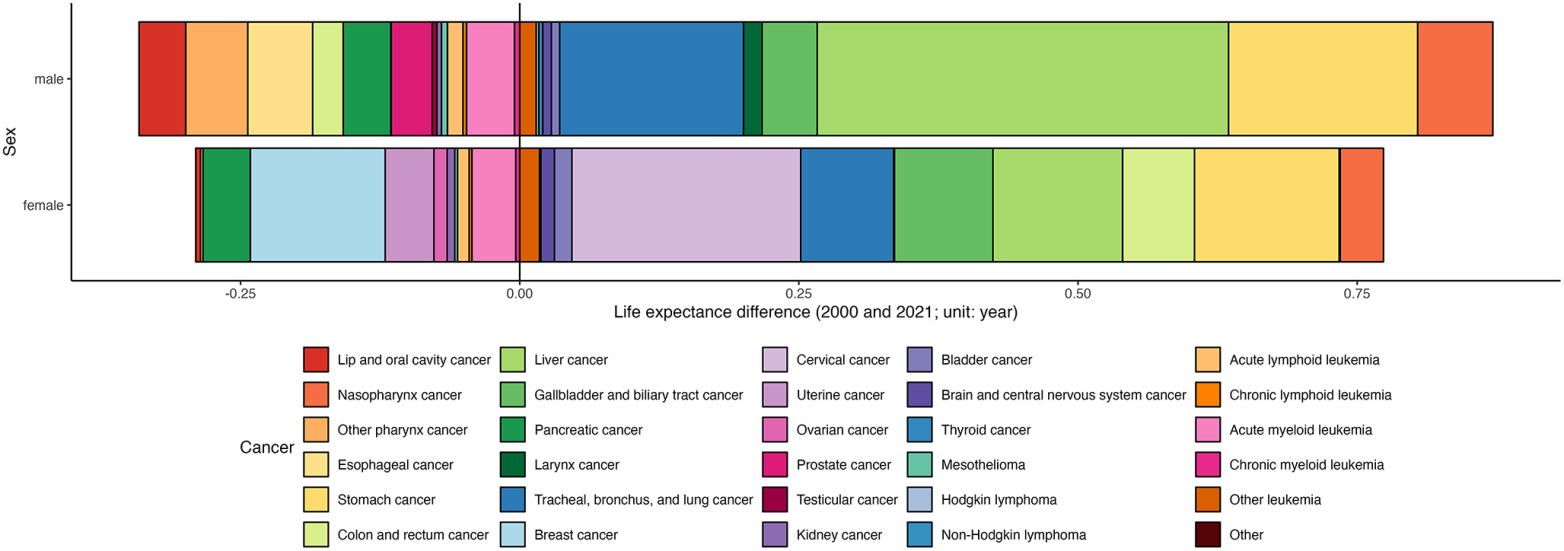
Comparison of life expectancy contributions for different cancer types between 2021 and 2000 for both sexes in Taiwan

The life expectancy decomposition from 2000 to 2021, attributable to specific cancer sites, revealed different contribution patterns for both sexes (Fig. 3). For female participants, gains in life expectancy were 0.20 years for cervical cancer, 0.13 years for stomach cancer, 0.12 years for liver cancer, 0.09 years for gallbladder and biliary tract cancer, 0.08 years for lung cancer, 0.06 years for colon and rectal cancer, and 0.04 years for nasopharynx cancer. By contrast, decreases in life expectancy observed for female participants were − 0.12 years for breast cancer, − 0.04 years for uterine cancer, and − 0.04 years for pancreatic cancer (Figure S9). For male participants, gains in life expectancy were 0.37 years for liver cancer, 0.17 years for stomach cancer, 0.16 years for lung cancer, 0.07 years for nasopharynx cancer, and 0.05 years for gallbladder and biliary tract cancer. By contrast, decreases in life expectancy observed for male participants were − 0.06 years for esophageal cancer, − 0.06 years for other pharyngeal cancer, − 0.04 years for acute myeloid leukemia, − 0.04 years for pancreatic cancer, and − 0.04 years for oral cancer (Figure S10).

## Discussion

Our analysis of the Taiwan Cancer Registry and National Death Registry data from 2000 to 2021 found that, age-standardized DALY rates decreased most likely due to improved screening, treatment, and risk reduction efforts [15–19]. The leading cancers by DALYs rates in 2021 were, lung, breast, liver, colorectal, and oral cancers as compared to liver, lung, colorectal, breast, and stomach cancer in 2000. Age-standardized DALY rates for lung and liver cancers significantly declined from 2000 to 2021, while colorectal and oral cancers remained stable and continued to be among the leading contributors to cancer burden. In contrast, the burden of breast cancer steadily increased over time. Liver cancer was the leading cause of cancer burden from 2000 to 2012, but lung cancer overtook it as the top burden in 2013. By 2021, breast cancer had surpassed liver cancer to become the second highest burden cancer. Breast cancer accounted for 19.5% of cancer-related DALYs among women, largely driven by premature deaths, while lung cancer was the leading cause of DALYs among men. Overall, mortality contributed more to the total cancer burden than morbidity. By 2021, reductions in lung, colorectal, and liver cancer mortality improved life expectancy, yet the increasing breast cancer burden had the opposite effect, possibly due to younger onset and rising mortality rates. Our life expectancy decomposition analysis revealed that shifts toward younger cancer onset are impacting population health. Addressing premature cancer-related deaths remains crucial for improving life expectancy outcomes.

Our findings indicated that breast and cervical cancers resulted in a higher average of years of life lost in the group of cancers with the highest mortality burden, demonstrating a specific pattern of age at death. An international comparison study reported a considerable difference in the age-specific incidence of breast cancer between Asian and Western women. The incidence rate of breast cancer among Taiwanese women aged ≤ 59 years has disproportionately increased over recent decades when compared with that in the United States, with the peak incidence occurring at the age of approximately 50 years in Taiwan versus approximately 70 years in the United States [20]. Health programs should focus on detecting cancers earlier and preventing the occurrence of disease when YLLs is the predominant concern. By contrast, health-care strategies should focus on reducing sequelae and enhancing quality of life when YLDs is the predominant concern. Moreover, the younger cancer population has a greater cancer burden when considering DALY/YLL estimates instead of mortality estimates. According to our 2021 estimates, 19.1% of all cancer-related DALYs and 8.1% of all cancer deaths occurred in the population aged below 50 years. In addition, DALYs are a useful tool for comparing health status and the overall burden across different causes within a population and for benchmarking population health against other countries or disease burden studies. To effectively use DALYs for assessing disease burden in Taiwan, it is crucial to continuously improve the accuracy and completeness of health data. Furthermore, implementing a monitoring and evaluation system can enable regular provision of longitudinal and cross-disease outcome comparisons, covering both communicable and noncommunicable diseases or exposures to modifiable risk factors [21]. This approach allows us to use such population health metrics as a comparative index for both within- and between-country comparisons.

Compared with the GBD study and other national disease burden studies, the main strength of the present study lies in the use of longitudinal individual data to provide detailed estimates of YLDs and prevalence for different cancer phases. Our study cohort obtained by linking data from the Taiwan Cancer Registry and National Death Registry enabled us to longitudinally estimate the overall cancer burden in Taiwan. According to the results of the 2021 GBD study, Taiwan had a crude prevalence of 3672.7 cancer cases per 100,000 population and a crude mortality rate of 235.5 per 100,000 population. The estimated DALYs from the GBD study were 5667.6 (95% uncertainty interval: 5260.4–5978.9) per 100,000 population, slightly higher than our estimates in the present study [1]. In GBD studies, data from cancer registries, vital statistics, and verbal autopsies are used to estimate cancer burden for 204 countries and territories. Previous research has indicated that the estimates of cancer burden in the GBD studies generally agree with estimates from the Global Cancer Incidence, Mortality, and Prevalence project [22]. However, our findings revealed a significantly lower crude prevalence rate estimate of 2680.9 per 100,000 population compared with GBD’s results. In addition, we found significant differences in the estimated burden of liver cancer between this study and the GBD results, with the respective age-standardized DALY estimates being 462.6 and 254.9 per 100,000 population (Table S4). Further verification revealed that the GBD’s estimate of liver cancer mortality in Taiwan significantly deviates from the official statistics released by the Taiwanese government. This discrepancy underscores the importance of using domestic health data for burden of disease estimation. Mattiuzzi and Lippi had compared cancer statistics between the global health estimates maintained by the World Health Organization and the GBD study in terms of DALYs and mortality for the 25 most common malignancies globally [23]. They found that cancer-related DALYs were highly similar but that cancer mortality rates were modestly but significantly overestimated in the GBD study. This discrepancy may be partly due to differences in data sources and estimation methods used. One specific factor is the redistribution process for noninformative or miscoded diseases (often referred to as “garbage codes”) in mortality estimates. In GBD studies, these garbage codes are routinely reassigned to more likely underlying causes of death on the basis of a predetermined list and evidence-based algorithms. However, a recent analysis examining the redistribution of garbage code deaths in Italy and Western Europe observed a wide heterogeneity in the pattern of garbage codes across European countries. This finding suggests differences in reporting behavior on death certificates and emphasizes the limitations and deficiencies of relying on a single algorithm–based approach [24]. Our model for the redistribution of garbage codes considered information on multiple causes of death and demographic characteristics to assign garbage codes more accurately and reduce the likelihood of misclassification [12].

This study has some limitations. First, although the completeness of the cancer registry database suggests the high representativeness of our data—reaching over 92% in 2002 and 97% since 2006—we might have missed a small number of cancer cases [11] Second, the DWs used in the present study were based on the 2021 GBD study. However, the methodological design and validity of DWs from the GBD study have faced criticism. In particular, the universality of these DWs has been questioned, although Salomon et al. reported a high degree of consistency in DW estimates between the 2010 GBD web-based surveys and the 2013 GBD European surveys [25]. The use of common DWs in YLD estimation may allow us to compare our estimates with other GBD studies; however, the choice of DW may affect results in terms of the effect size of YLDs and even cancer ranking. In our study, the YLD component accounted for a relatively small proportion of DALYs for most cancer groups. Thus, we believe that the effect of any inaccuracies in DW estimates should be limited. Third, the estimates of cancer burden in our study may be biased because the values of cure time were adopted from the GBD study. Empirical data suggest that time-to-cure varies substantially across different periods, cancer types, and countries [26]. Furthermore, other assumptions used in calculating DALYs, such as the duration of cancer phases, were derived from the GBD study, consistent with methodologies used in other burden of disease studies [5, 7, 27]. This consistency allowed us to compare between studies. However, we still lack detailed records of disease progression at the individual level due to the absence of data on disease recurrence and treatment details in the registry system. Consequently, the results the non-fatal burden may be underestimated. Finally, in our analysis of the contribution to life expectancy, we did not account for factors such as health-care quality and access, socioeconomic variables, and other relevant parameters that can substantially affect outcomes. We considered mortality as an outcome influenced by multiple factors. However, our focus was solely on mortality as a factor affecting life expectancy, limiting our scope and excluding other factors that could also influence cancer mortality. Thus, our interpretation might not be applicable to other specific factors that potentially affect cancer mortality.

## Conclusion

The present study provides an overview of the epidemiological landscape of cancer burden in Taiwan by analyzing morbidity, mortality, and adjusted disability for 30 cancer groups. In the Taiwanese population, lung cancer represents the highest burden, followed by breast, liver, colorectal, and lip and oral cavity cancers. The development of effective cancer control strategies requires careful consideration of both the local health infrastructure and disease epidemiology. Given the changes in cancer burden over recent decades, active participation from all stakeholders, including health policymakers, health-care providers, and the general public, is imperative to collaboratively set priorities and implement evidence-based cancer control strategies.

## Electronic Supplementary Material

Below is the link to the electronic supplementary material.

Supplementary Material 1

## Data Availability

The data used in this analysis are not owned by the authors and therefore cannot be shared publicly. To acquire access to the individual-level data, interested researchers must complete an application form, submit a research proposal, and provide documentation of institutional review board approval to the Health and Welfare Data Science Center, Taiwan. The Center will review these materials and grant access to eligible researchers who meet the criteria for accessing confidential data. It should be noted that authorized researchers will be granted access to the data under the same conditions and procedures as the authors.
