# Supplementary material for: Disease Burden of 30 Cancer Groups in Taiwan from 2000 to 2021
Source: J Epidemiol Glob Health. 2025 Apr 22;15(1):62. doi: 10.1007/s44197-025-00406-w (PMC12014961; doi:10.1007/s44197-025-00406-w)
Supplement: Supplementary file 1 — Supplementary Material 1 [file 44197_2025_406_MOESM1_ESM.docx]

**Disease burden of 30 cancer groups in Taiwan from 2000 to 2021**

**Supplementary Methods and Results**

**Contents**

**Table S1.** ICD map for cancer sites.

**Table S2.** Duration of the cancer phases.

**Table S3.** Disability weights of the cancer phases.

**Table S4.** The comparison of age-standardized rate per 100,000 population for mortality, prevalence, years of life lost, years lived with disability, and disability-adjusted life years between GBD 2021 study and the present study for Taiwan in 2021

**Figure S1.** Mortality rate of 30 cancer types before (left panel) and after (right panel) garbage codes redistribution procedure.

**Figure S2.** Definition of cancer phases classification for non-fatal burden (YLD) estimation

**Figure S3.** Time trends of age-standardized incidence (per 100,000 population) for 30 cancer groups in Taiwan from 2000 to 2021.

**Figure S4.** Time trends of age-standardized prevalence (per 100,000 population) for 30 cancer groups in Taiwan from 2000 to 2021.

**Figure S5.** Time trends of age-standardized mortality rate (per 100,000 population) for 30 cancer groups in Taiwan from 2000 to 2021.

**Figure S6.** Time trends of age-standardized YLD rate (per 100,000 population) for 30 cancer groups in Taiwan from 2000 to 2021.

**Figure S7.** Time trends of age-standardized YLL rate (per 100,000 population) for 30 cancer groups in Taiwan from 2000 to 2021.

**Figure S8.** Time trends of age-standardized DALY rate (per 100,000 population) for 30 cancer groups in Taiwan from 2000 to 2021.

**Figure S9.** Decomposition of life expectancy contributions by cancer type in Taiwan, 2021 vs. 2000 (Female)

**Figure S10.** Decomposition of life expectancy contributions by cancer type in Taiwan, 2021 vs. 2000 (Male)

**Table S1.** ICD map for cancer sites.

| **Cause** | **ICD** | **ICD-O-3 Morphology** |
| --- | --- | --- |
| Uterine cancer | 182; C54 | NA |
| Thyroid cancer | 193; C73 | NA |
| Testicular cancer | 186; C62 | NA |
| Stomach cancer | 151; C16 | NA |
| Prostate cancer | 185; C61 | NA |
| Pancreatic cancer | 157; C25 | NA |
| Ovarian cancer | 183; C56 | NA |
| Other pharynx cancer | 146; 148; C09; C10; C12; C13 | NA |
| Other | C009; C029; C049; C069; C089; C099; C108; C119; C482; C319;  C329; C339; C349; C384; C383; C379; C380; C388; C410; C411;  C412; C413; C414; C400; C401; C402; C403; C419; C490; C491;  C492; C493; C494; C495; C496; C498; C499; C629; C609; C619;  C630; C632; C638; C649; C659; C669; C679; C688; C779; C529;  C569; C519; C578 | 80000 |
|  | C159; C169; C189; C209; C211; C210; C218; C249; C329; C339;  C349; C509; C609; C619; C679; C699; C759; C539; C548; C579 | 80102 |
|  | C424 | 97401 |
|  | C729 | 95401 |
|  | C424 | 99503 |
|  | C480; C481; C809; C509; C559; C569; C739 | 80003 |
|  | C089; C482; C329; C349; C384; C629; C619; C679; C689; C751;  C753; C749; C755; C719; C700; C419; C499; C509; C809; C589;  C569; C729 | 80001 |
| Other leukemia | NA | 98203; 98323; 98603 |
| Lip and oral cavity cancer | 140; 141; 142; 143; 144; 145;  C00; C01; C02; C03; C04; C05; C06; C07; C08 | NA |
| Non-Hodgkin lymphoma | NA | 96893; 96993; 96713; 97613; 96753;  96903; 96913; 96953; 96983; 96733;  96873; 98263; 96803; 96843;  96793; 95963; 96703; 96783; 97283;  98333; 99403; 97193; 97003; 97013;  97093; 97183; 97173; 97163; 97083;  97023; 97053; 97143; 98273; 97293;  98343; 99483; 97313; 97323; 97333;  97343 |
| Nasopharynx cancer | 147; C11 | NA |
| Mesothelioma | NA | 905 |
| Tracheal, bronchus, and lung cancer | 162; C33; C34 | NA |
| Liver cancer | 155; C22 | NA |
| Larynx cancer | 161; C32 | NA |
| Kidney cancer | 189; C64; C65; C66; C68; C69 | NA |
| Hodgkin lymphoma | NA | 96503; 96513; 96523; 96533; 96593;  96613; 96623; 96633; 96643; 96653;  96673 |
| Gallbladder and biliary tract cancer | 156; C23; C24 | NA |
| Esophageal cancer | 150; C15 | NA |
| Colon and rectum cancer | 153; 154; C18; C19; C20; C21 | NA |
| Chronic myeloid leukemia | NA | 98753 |
| Chronic lymphoid leukemia | NA | 98233 |
| Cervical cancer | 180; C53; D26 | NA |
| Breast cancer | 174; 175; C50 | NA |
| Brain and central nervous system cancer | 191; 192; C70; C71; C72 | NA |
| Bladder cancer | 188; C67 | NA |
| Acute myeloid leukemia | NA | 98663; 98713; 98963; 98973; 98953;  99203; 98613; 98403; 98673; 98703;  98723; 98733; 98743; 98913; 99103;  99313; 99303; 97273 |
| Acute lymphoid leukemia | NA | 98353; 98363; 98373 |

**Table S2.** Duration of the cancer phases.

| Cause | Diagnosis and treatment  (month) | Metastatic  (month) | Terminal  (month) |
| --- | --- | --- | --- |
| Esophageal cancer | 5 | 4.6 | 1 |
| Stomach cancer | 5.2 | 3.88 | 1 |
| Liver cancer | 4 | 2.51 | 1 |
| Larynx cancer | 5.3 | 8.84 | 1 |
| Tracheal, bronchus, and lung cancer | 3.3 | 4.51 | 1 |
| Breast cancer | 3 | 17.7 | 1 |
| Cervical cancer | 4.8 | 9.21 | 1 |
| Uterine cancer | 4.6 | 11.6 | 1 |
| Prostate cancer | 4 | 30.35 | 1 |
| Colon and rectum cancer | 4 | 9.69 | 1 |
| Lip and oral cavity cancer | 5.3 | 9.33 | 1 |
| Nasopharynx cancer | 5.3 | 13.19 | 1 |
| Other pharynx cancer | 5.3 | 7.91 | 1 |
| Gallbladder and biliary tract cancer | 4 | 3.47 | 1 |
| Pancreatic cancer | 4.1 | 2.54 | 1 |
| Ovarian cancer | 3.2 | 25.6 | 1 |
| Testicular cancer | 3.7 | 19.47 | 1 |
| Kidney cancer | 5.3 | 5.38 | 1 |
| Bladder cancer | 5.1 | 5.8 | 1 |
| Brain and central nervous system cancer | 5 | 6.93 | 1 |
| Thyroid cancer | 3 | 19.39 | 1 |
| Mesothelioma | 4 | 7.75 | 1 |
| Hodgkin lymphoma | 3.7 | 26 | 1 |
| Non-Hodgkin lymphoma | 3.7 | 7.7 | 1 |
| Acute lymphoid leukemia | 12 | 7.02 | 1 |
| Acute myeloid leukemia | 6 | 4.6 | 1 |
| Chronic lymphoid leukemia | 6 | 48 | 1 |
| Chronic myeloid leukemia | 6 | 4.6 | 1 |
| Other leukemia | 6 | 48 | 1 |
| Other | 4.4 | 15.81 | 1 |

**Table S3.** Disability weights of the cancer phases.

| **Phases** | **Disability weights** |
| --- | --- |
| **Diagnosis and treatment** | 0.288 |
| **Remission** | 0.049 |
| **Metastatic** | 0.451 |
| **Terminal** | 0.540 |

**Table S4.** The comparison of age-standardized rate per 100,000 population for mortality, prevalence, years of life lost, years lived with disability, and disability-adjusted life years between GBD 2021 study and the present study for Taiwan in 2021

|  |  | **Age-standardized rate (per 100,000 population) for both sexes in 2021** | | | | | | | | | | | | | | | | | | | | | | | | | |  |
| --- | --- | --- | --- | --- | --- | --- | --- | --- | --- | --- | --- | --- | --- | --- | --- | --- | --- | --- | --- | --- | --- | --- | --- | --- | --- | --- | --- | --- |
|  |  | **Mortality** | | | | **Years of life lost** | | | | | | **Prevalence** | | | | | **Years lived with disability** | | | | | | | **Disability-adjusted life years** | | | | |
| **Cancer groups/year** |  | GBD 2021 | present study | Difference  (rate; %) | | GBD 2021 | present study | | Difference  (rate; %) | | | GBD 2021 | present study | Difference  (rate; %) | | | GBD 2021 | | present study | | Difference  (rate; %) | | | GBD 2021 | present study | Difference  (rate; %) | | |
| Tracheal, bronchus, and lung cancer |  | 27.02 | 27.33 | -0.31 | -1.12% | 595.09 | 559.4 | 35.7 | | | 6.38% | 36.8 | 149.3 | -112.5 | -75.35% | | | 6.75 | | 13.91 | | -7.16 | -51.47% | 601.84 | 573.3 | 28.54 | 4.98% | |
| Breast cancer* |  | 13.28 | 15.63 | -2.35 | -15.06% | 407.4 | 451.75 | -44.34 | | | -9.82% | 560.86 | 598.21 | -37.35 | -6.24% | | | 39.2 | | 42.11 | | -2.92 | -6.92% | 446.6 | 493.86 | -47.26 | -9.57% | |
| Liver cancer |  | 9.84 | 21.06 | -11.21 | -53.26% | 252.17 | 453.29 | -201.12 | | | -44.37% | 17.08 | 110.37 | -93.29 | -84.52% | | | 2.76 | | 9.34 | | -6.58 | -70.47% | 254.93 | 462.63 | -207.7 | -44.90% | |
| Colon and rectum cancer |  | 21.86 | 18.34 | 3.52 | 19.19% | 477.07 | 378.84 | 98.23 | | | 25.93% | 296.76 | 261.24 | 35.53 | 13.60% | | | 26.43 | | 21.55 | | 4.87 | 22.62% | 503.5 | 400.39 | 103.1 | 25.75% | |
| Lip and oral cavity cancer |  | 5.57 | 6.75 | -1.18 | -17.43% | 170.65 | 197.94 | -27.29 | | | -13.78% | 85.07 | 106.18 | -21.11 | -19.88% | | | 7.46 | | 9.4 | | -1.94 | -20.68% | 178.11 | 207.34 | -29.23 | -14.10% | |
| Esophageal cancer |  | 7.45 | 5.49 | 1.96 | 35.76% | 216.66 | 159.08 | 57.58 | | | 36.20% | 21.51 | 20.05 | 1.46 | 7.29% | | | 2.89 | | 2.54 | | 0.35 | 13.81% | 219.55 | 161.62 | 57.93 | 35.84% | |
| Prostate cancer* |  | 10.83 | 10.39 | 0.44 | 4.22% | 167.88 | 136.63 | 31.26 | | | 22.88% | 292.64 | 252.93 | 39.71 | 15.70% | | | 24.19 | | 21.57 | | 2.62 | 12.16% | 192.07 | 158.19 | 33.88 | 21.42% | |
| Pancreatic cancer |  | 7.1 | 7.14 | -0.04 | -0.56% | 166.52 | 156.04 | 10.48 | | | 6.72% | 6.19 | 13.33 | -7.14 | -53.57% | | | 1.47 | | 1.9 | | -0.42 | -22.32% | 167.99 | 157.94 | 10.06 | 6.37% | |
| Stomach cancer |  | 8.86 | 6.22 | 2.64 | 42.45% | 190.56 | 127.05 | 63.51 | | | 49.99% | 23.94 | 44.4 | -20.45 | -46.07% | | | 3.14 | | 3.96 | | -0.82 | -20.66% | 193.7 | 131.01 | 62.69 | 47.85% | |
| Ovarian cancer* |  | 3.19 | 3.68 | -0.49 | -13.34% | 97.16 | 107.27 | -10.11 | | | -9.42% | 53.4 | 62.11 | -8.71 | -14.03% | | | 5.86 | | 5.65 | | 0.21 | 3.76% | 103.02 | 112.92 | -9.89 | -8.76% | |
| Uterine cancer* |  | 1.34 | 2.96 | -1.62 | -54.62% | 37.7 | 83.29 | -45.59 | | | -54.73% | 86.32 | 109.63 | -23.31 | -21.27% | | | 5.68 | | 7.68 | | -2 | -26.01% | 43.38 | 90.96 | -47.58 | -52.31% | |
| Other pharynx cancer |  | 2.26 | 2.78 | -0.53 | -18.89% | 69.05 | 82.36 | -13.31 | | | -16.16% | 12.41 | 25.03 | -12.63 | -50.44% | | | 1.64 | | 2.43 | | -0.79 | -32.46% | 70.69 | 84.79 | -14.1 | -16.63% | |
| Non-Hodgkin lymphoma |  | 3.62 | 3.62 | 0.01 | 0.17% | 94.87 | 79.1 | 15.77 | | | 19.94% | 41.76 | 41.87 | -0.1 | -0.25% | | | 4.57 | | 3.32 | | 1.25 | 37.66% | 99.44 | 82.42 | 17.02 | 20.65% | |
| Cervical cancer* |  | 3.67 | 2.79 | 0.88 | 31.49% | 102.13 | 73.78 | 28.35 | | | 38.42% | 70.6 | 53.64 | 16.97 | 31.63% | | | 5.41 | | 4.07 | | 1.34 | 32.91% | 107.54 | 77.85 | 29.69 | 38.13% | |
| Brain and central nervous system cancer |  | 1.93 | 1.94 | -0.01 | -0.67% | 70.81 | 74.95 | -4.14 | | | -5.52% | 11.92 | 13.45 | -1.54 | -11.44% | | | 1.27 | | 1.32 | | -0.05 | -4.12% | 72.07 | 76.27 | -4.19 | -5.50% | |
| Kidney cancer |  | 2.76 | 2.41 | 0.35 | 14.57% | 64.87 | 54.96 | 9.92 | | | 18.04% | 56.73 | 49.97 | 6.76 | 13.53% | | | 4.18 | | 3.67 | | 0.51 | 13.94% | 69.05 | 58.62 | 10.43 | 17.79% | |
| Nasopharynx cancer |  | 1.99 | 1.82 | 0.16 | 9.00% | 64.46 | 55.17 | 9.29 | | | 16.85% | 22.6 | 32.6 | -10 | -30.67% | | | 2.22 | | 2.72 | | -0.51 | -18.70% | 66.68 | 57.89 | 8.78 | 15.17% | |
| Bladder cancer |  | 3.2 | 3.01 | 0.19 | 6.31% | 57.83 | 48.32 | 9.51 | | | 19.69% | 54.23 | 34.66 | 19.57 | 56.47% | | | 4.95 | | 2.75 | | 2.2 | 79.87% | 62.79 | 51.07 | 11.71 | 22.93% | |
| Acute myeloid leukemia |  | 2.2 | 1.67 | 0.53 | 31.77% | 73.54 | 48.55 | 24.98 | | | 51.45% | 3.35 | 7.56 | -4.21 | -55.68% | | | 0.69 | | 0.7 | | -0.02 | -2.19% | 74.22 | 49.25 | 24.97 | 50.70% | |
| Gallbladder and biliary tract cancer |  | 2.15 | 1.66 | 0.49 | 29.75% | 46.9 | 32.78 | 14.12 | | | 43.07% | 4.92 | 7.74 | -2.82 | -36.46% | | | 0.8 | | 0.78 | | 0.02 | 2.50% | 47.7 | 33.56 | 14.14 | 42.13% | |
| Other leukemia |  | 0.25 | 0.94 | -0.69 | -73.08% | 6.87 | 23.24 | -16.38 | | | -70.45% | 2.17 | 0.03 | 2.14 | 6119.95% | | | 0.26 | | 0 | | 0.26 | -- | 7.13 | 23.24 | -16.11 | -69.32% | |
| Acute lymphoid leukemia |  | 0.44 | 0.4 | 0.04 | 9.65% | 21.26 | 19.63 | 1.63 | | | 8.31% | 7.57 | 1.97 | 5.6 | 284.54% | | | 0.7 | | 0.18 | | 0.52 | 282.94% | 21.96 | 19.8 | 2.15 | 10.87% | |
| Thyroid cancer |  | 0.54 | 0.59 | -0.06 | -9.50% | 12.64 | 11.17 | 1.47 | | | 13.17% | 50.18 | 111.39 | -61.2 | -54.95% | | | 2.94 | | 6.33 | | -3.39 | -53.49% | 15.58 | 17.5 | -1.92 | -10.95% | |
| Larynx cancer |  | 0.59 | 0.69 | -0.1 | -14.04% | 14.6 | 15.33 | -0.74 | | | -4.79% | 10.67 | 11.86 | -1.19 | -10.06% | | | 1.02 | | 0.92 | | 0.11 | 11.52% | 15.62 | 16.25 | -0.63 | -3.87% | |
| Testicular cancer* |  | 0.14 | 0.19 | -0.05 | -27.46% | 5.58 | 9.77 | -4.19 | | | -42.87% | 21.1 | 20.39 | 0.71 | 3.47% | | | 1.29 | | 1.25 | | 0.04 | 3.05% | 6.87 | 11.02 | -4.15 | -37.66% | |
| Chronic myeloid leukemia |  | 0.21 | 0.18 | 0.03 | 16.28% | 6.11 | 4.26 | | | 1.85 | 43.49% | 0.95 | 5.94 | -4.99 | | -83.96% | 0.13 | | 0.4 | | -0.26 | | -66.25% | 6.25 | 4.66 | 1.58 | 33.91% | |
| Mesothelioma |  | 0.11 | 0.18 | -0.07 | -38.01% | 3.18 | 3.93 | | | -0.74 | -18.92% | 0.23 | 0.58 | -0.35 | | -60.43% | 0.04 | | 0.1 | | -0.05 | | -54.17% | 3.23 | 4.02 | -0.8 | -19.77% | |
| Chronic lymphoid leukemia |  | 0.17 | 0.14 | 0.03 | 21.19% | 4.11 | 2.26 | | | 1.85 | 81.48% | 4.16 | 3.78 | 0.39 | | 10.27% | 0.5 | | 0.33 | | 0.17 | | 52.48% | 4.61 | 2.6 | 2.01 | 77.17% | |
| Hodgkin lymphoma |  | 0.04 | 0.04 | 0.01 | 15.68% | 1.47 | 1.07 | | | 0.4 | 37.99% | 2.93 | 6.66 | -3.73 | | -56.02% | 0.23 | | 0.39 | | -0.16 | | -40.63% | 1.7 | 1.46 | 0.24 | 16.72% | |
| Other |  | 5.57 | 6.31 | -0.74 | -11.79% | 150.3 | 154.93 | | | -4.62 | -2.98% | 60.09 | 95.79 | -35.69 | | -37.26% | 5.75 | | 6.84 | | -1.09 | | -15.93% | 156.05 | 161.76 | -5.71 | -3.53% | |
| Total |  | 148.19 | 156.35 | -8.16 | -5.22% | 3,649.45 | 3,606.11 | | | 43.34 | 1.20% | 1,919.14 | 2,252.66 | -333.51 | | -14.81% | 164.4 | | 178.09 | | -13.69 | | -7.69% | 3,813.86 | 3,784.20 | 29.65 | 0.78% | |

*Rates for sex-specific cancer types were calculated using only the female population for cervical, ovarian, uterine, and breast cancers and only the male population for prostate and testicular cancers.


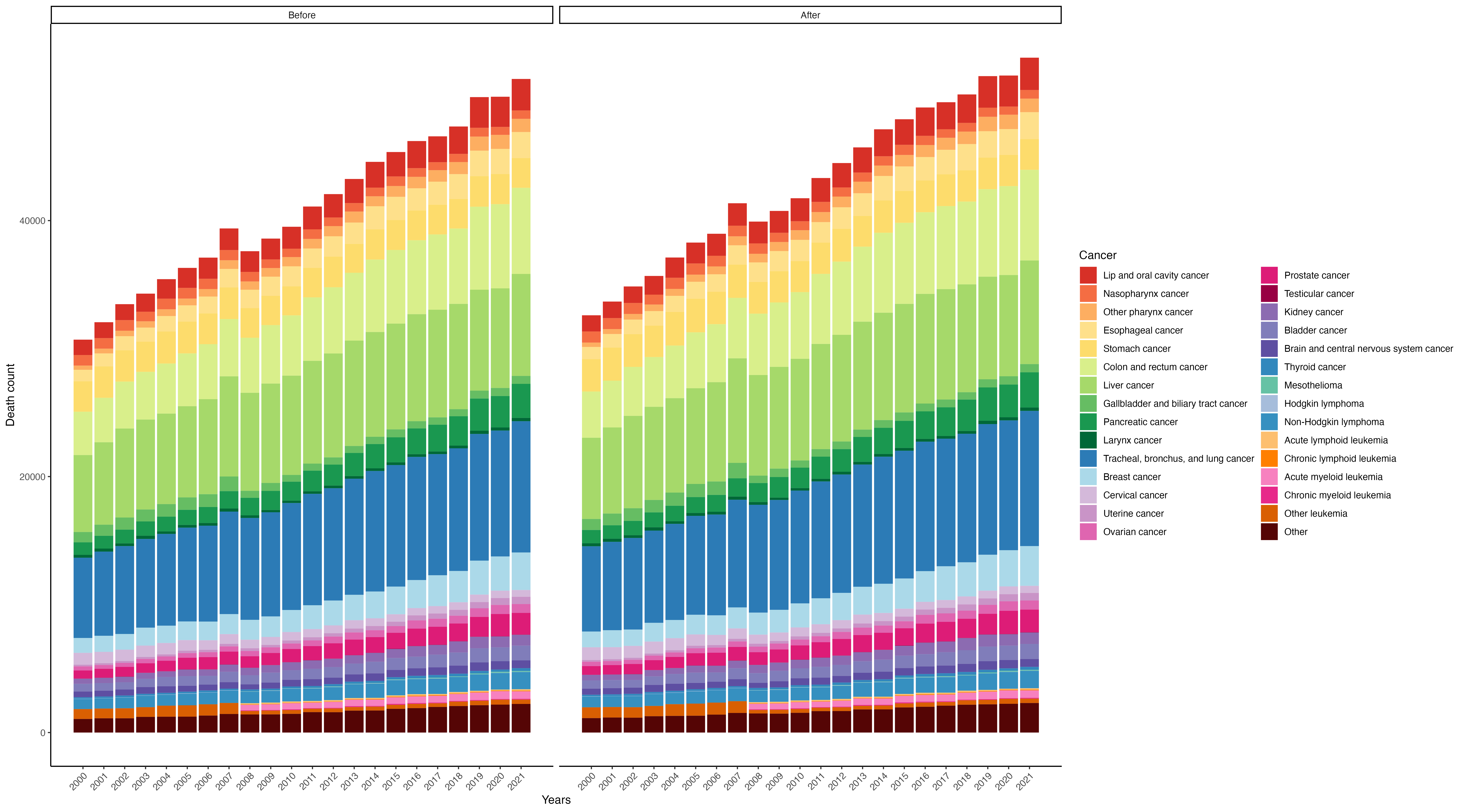


**Figure S1.** Mortality rate of 30 cancer types before (left panel) and after (right panel) garbage codes redistribution procedure.


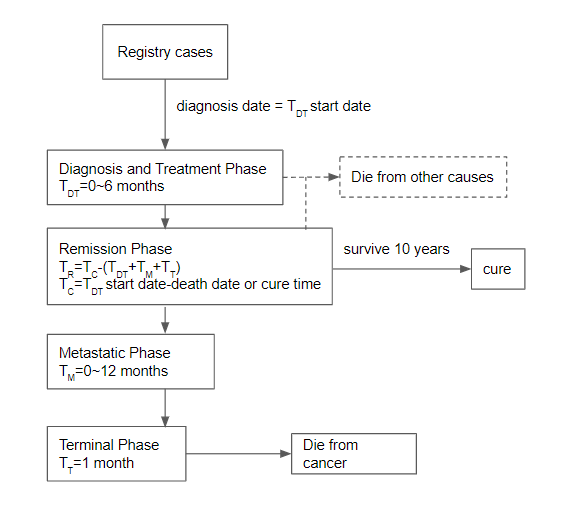
**Figure S2.** Definition of cancer phases classification for non-fatal burden (YLD) estimation. $T_{DT}$ : Duration of diagnosis and treatment phase. $T_{R}$ : Duration of remission phase. $T_{C}$ : Duration of hypothetical cure phase. $T_{M}$ : Duration of metastatic. $T_{T}$ : Duration of terminal phases. We used the column “DIAG_DT” from Taiwan Cancer Registration database as the $T_{DT}$ starting date. The end date of $T_{T}$ was the death date in the Taiwan Vital Registration database.


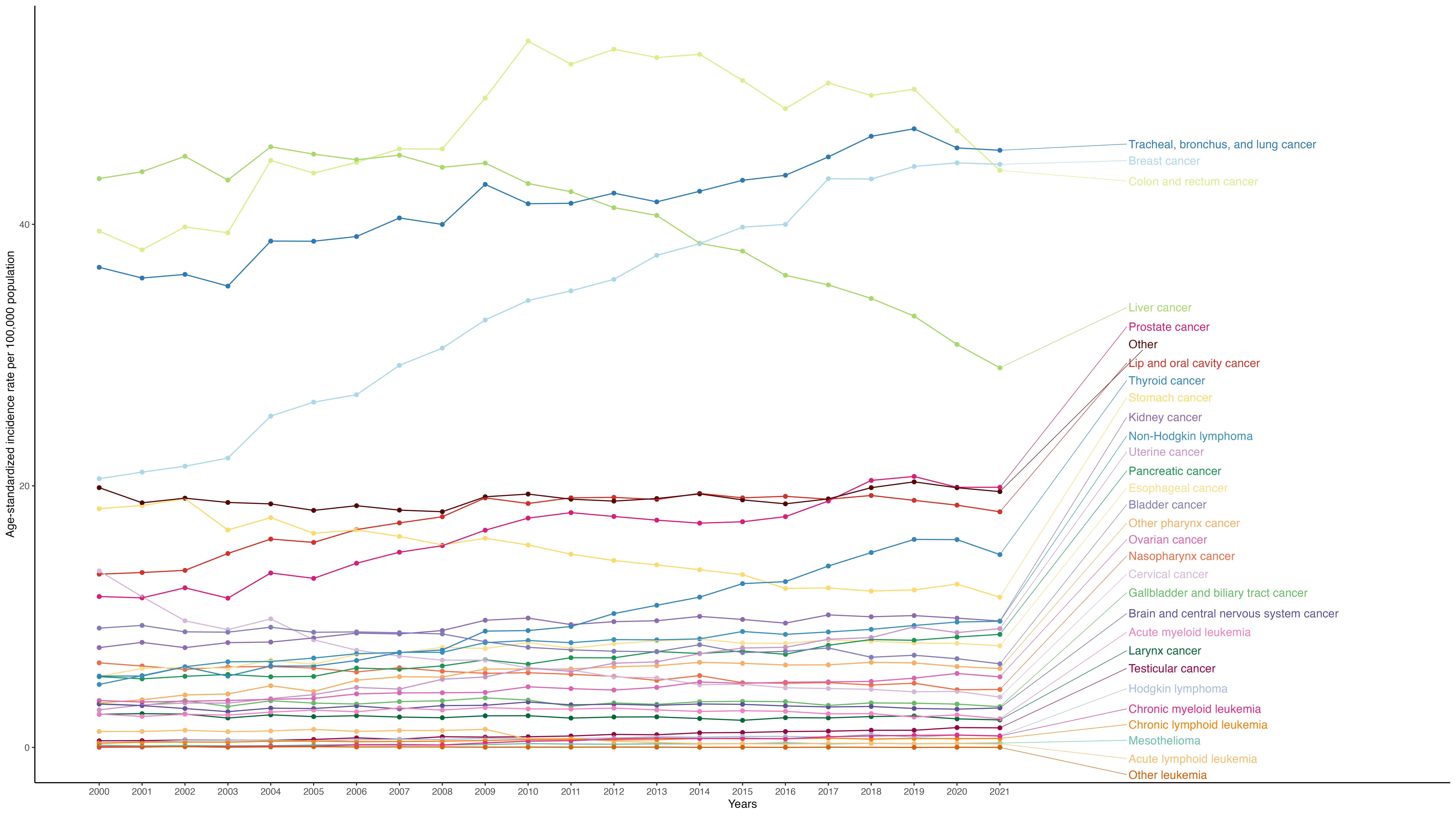


**Figure S3.** Time trends of age-standardized incidence (per 100,000 population) for 30 cancer groups in Taiwan from 2000 to 2021.


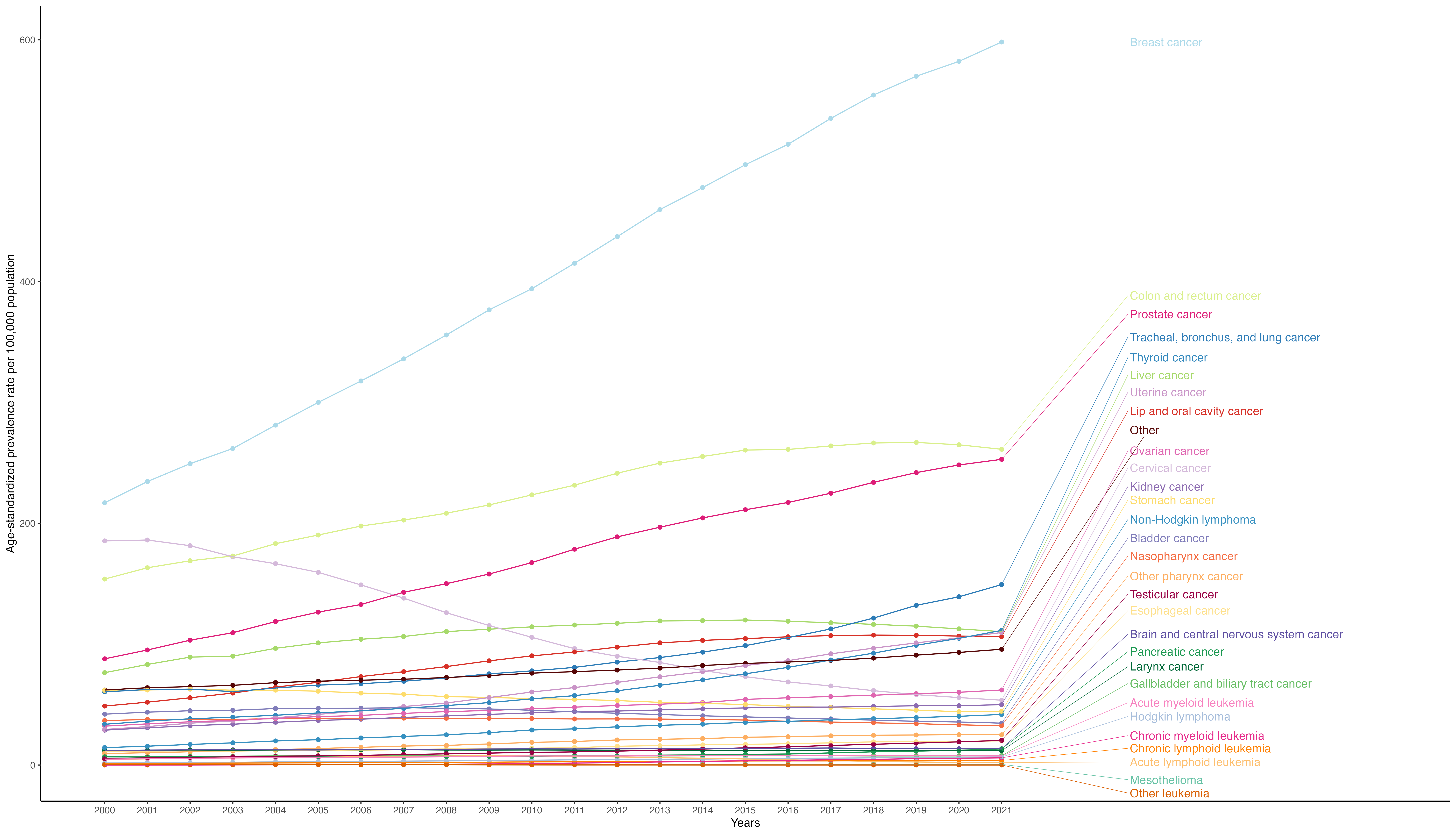


**Figure S4.** Time trends of age-standardized prevalence (per 100,000 population) for 30 cancer groups in Taiwan from 2000 to 2021.


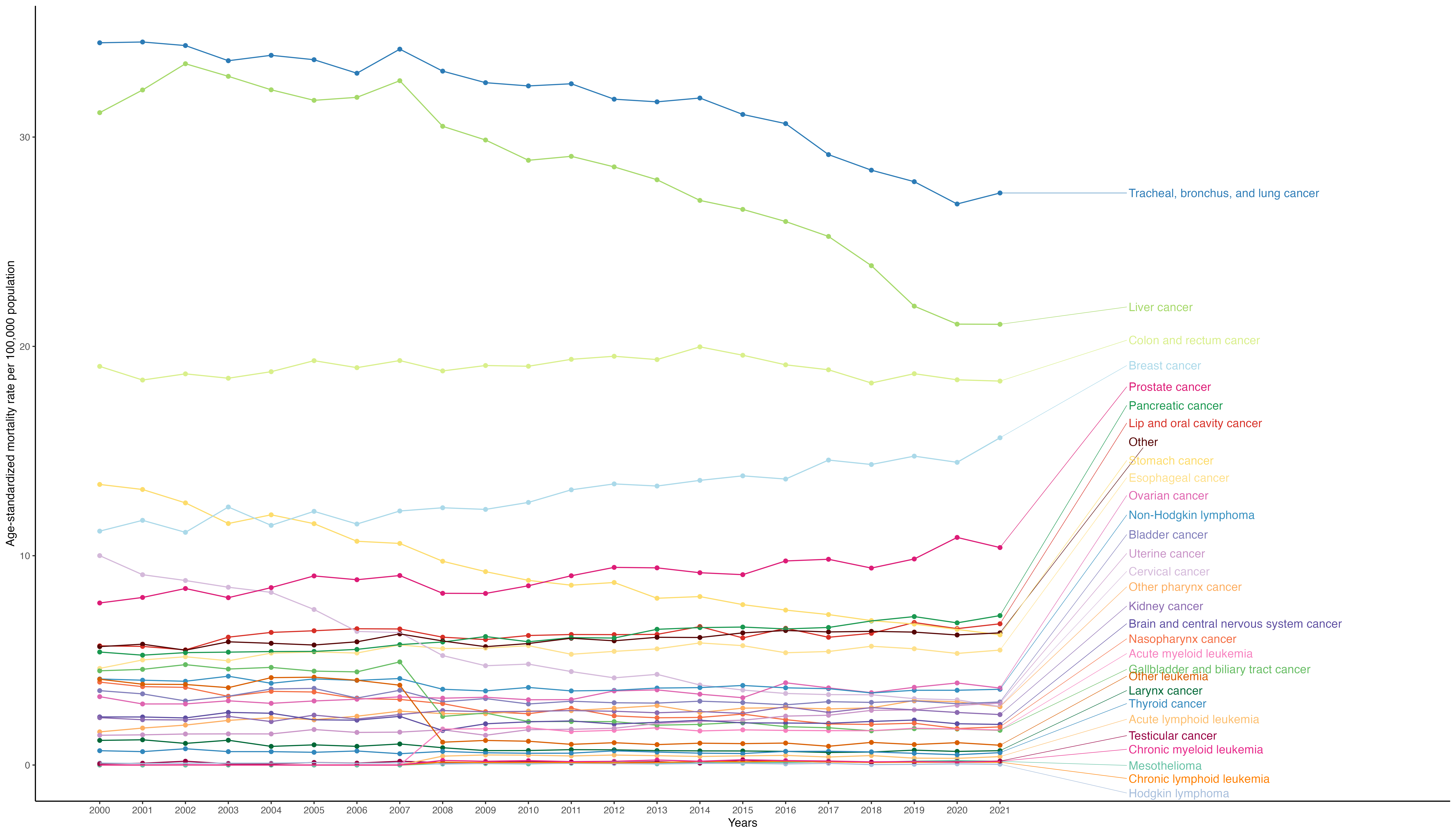


**Figure S5.** Time trends of age-standardized mortality rate (per 100,000 population) for 30 cancer groups in Taiwan from 2000 to 2021.


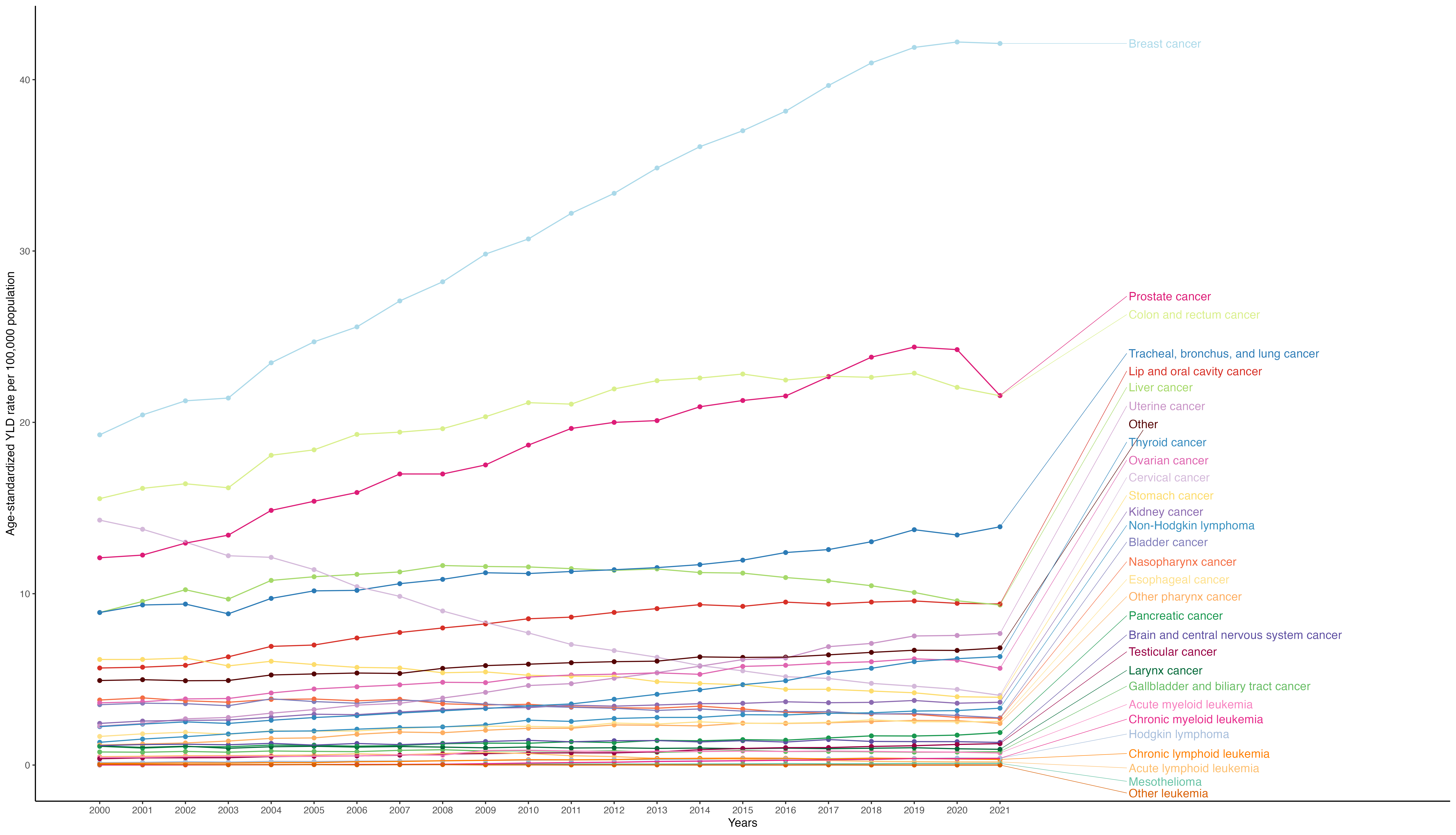


**Figure S6.** Time trends of age-standardized YLD rate (per 100,000 population) for 30 cancer groups in Taiwan from 2000 to 2021.


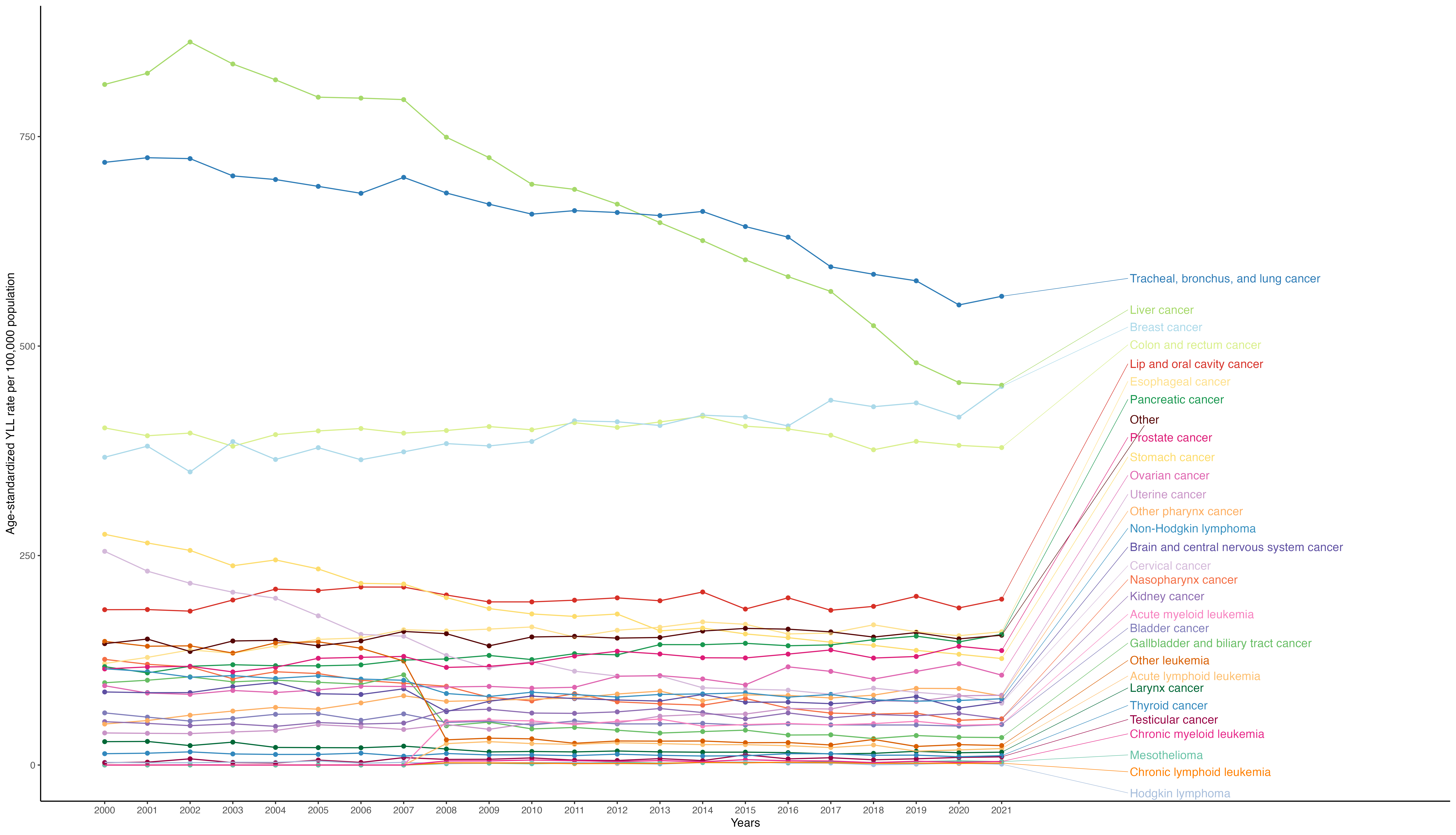


**Figure S7.** Time trends of age-standardized YLL rate (per 100,000 population) for 30 cancer groups in Taiwan from 2000 to 2021.


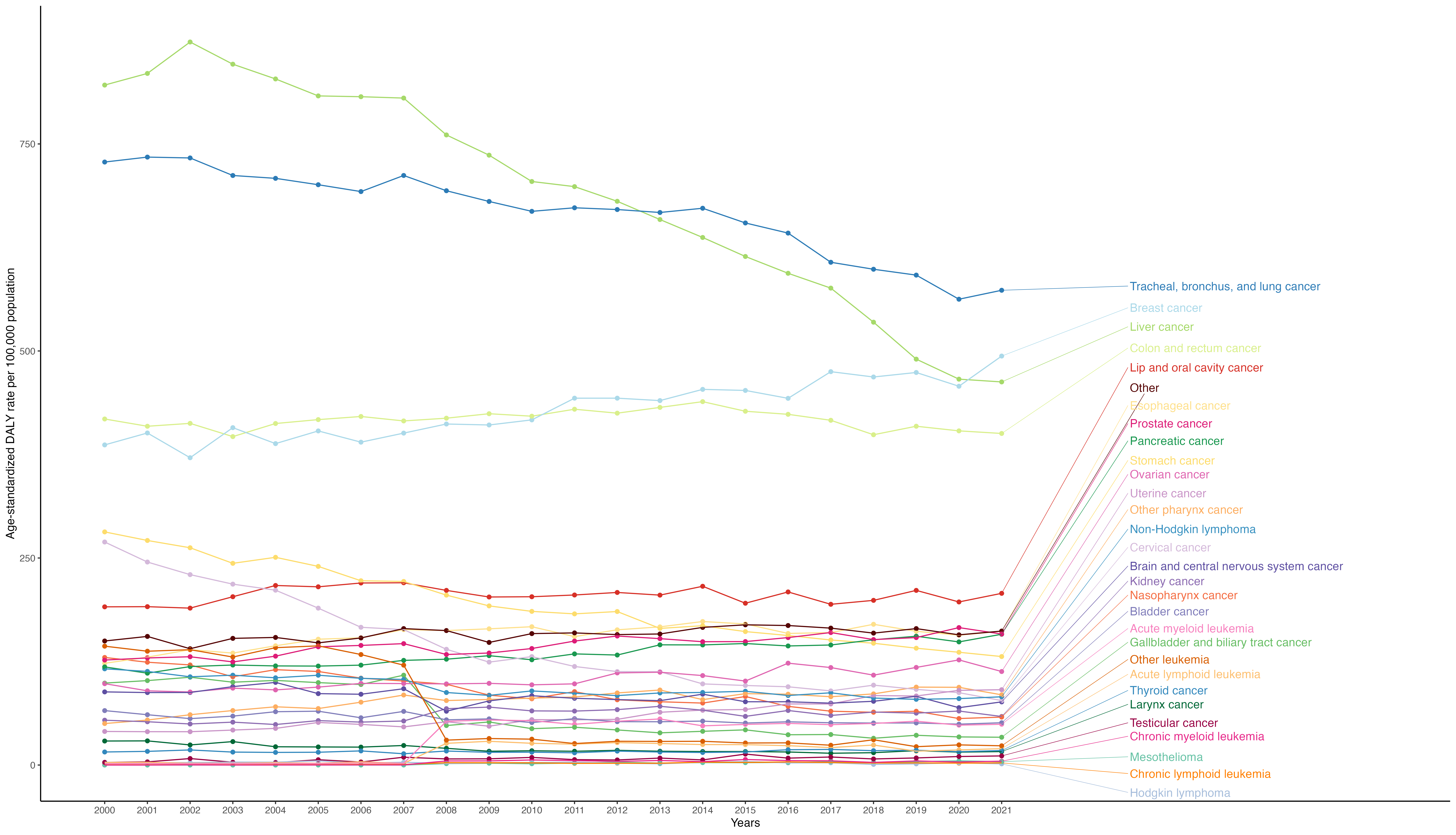


**Figure S8.** Time trends of age-standardized DALY rate (per 100,000 population) for 30 cancer groups in Taiwan from 2000 to 2021.


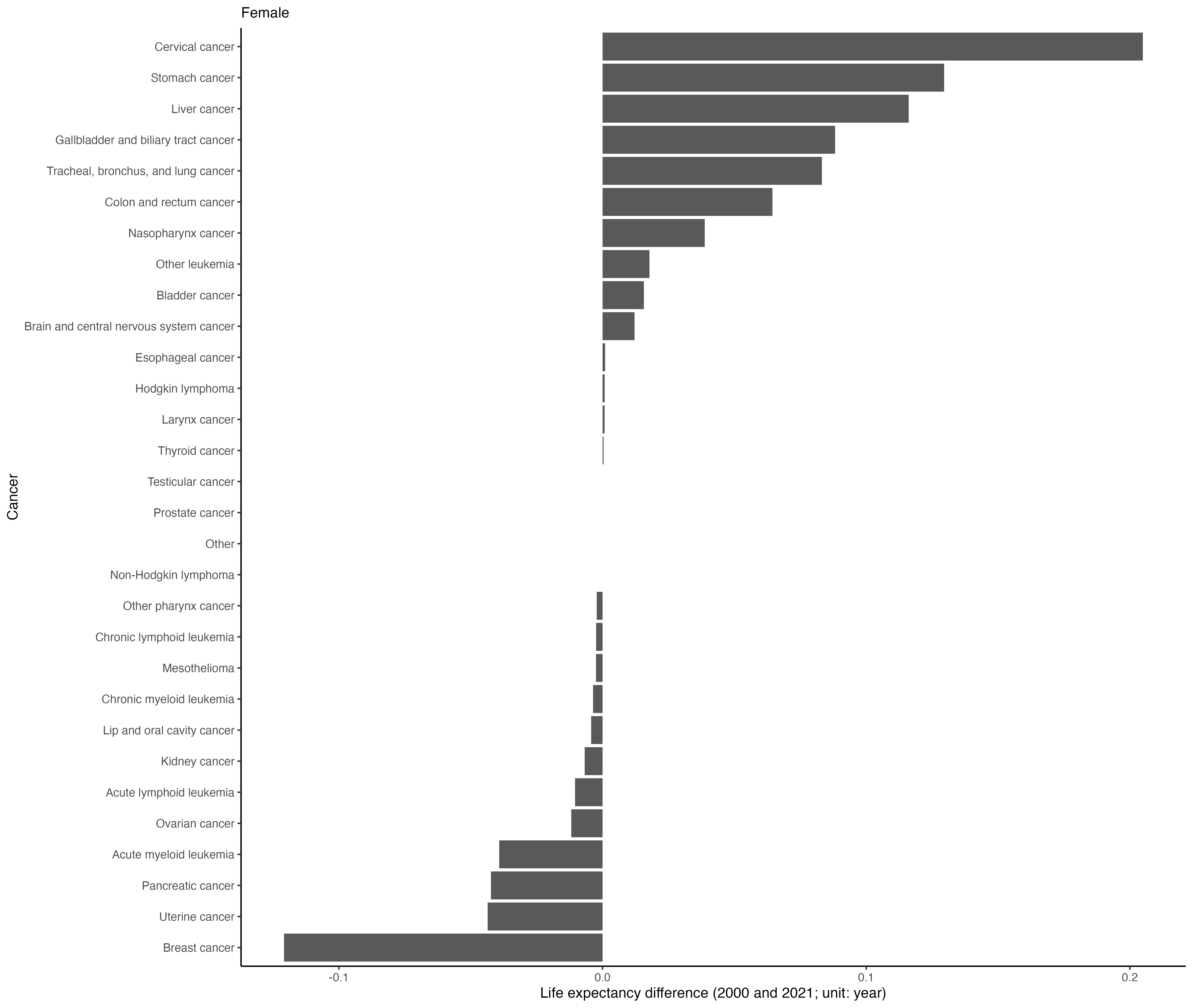


**Figure S9.** Decomposition of life expectancy contributions by cancer type in Taiwan, 2021 vs. 2000 (Female).


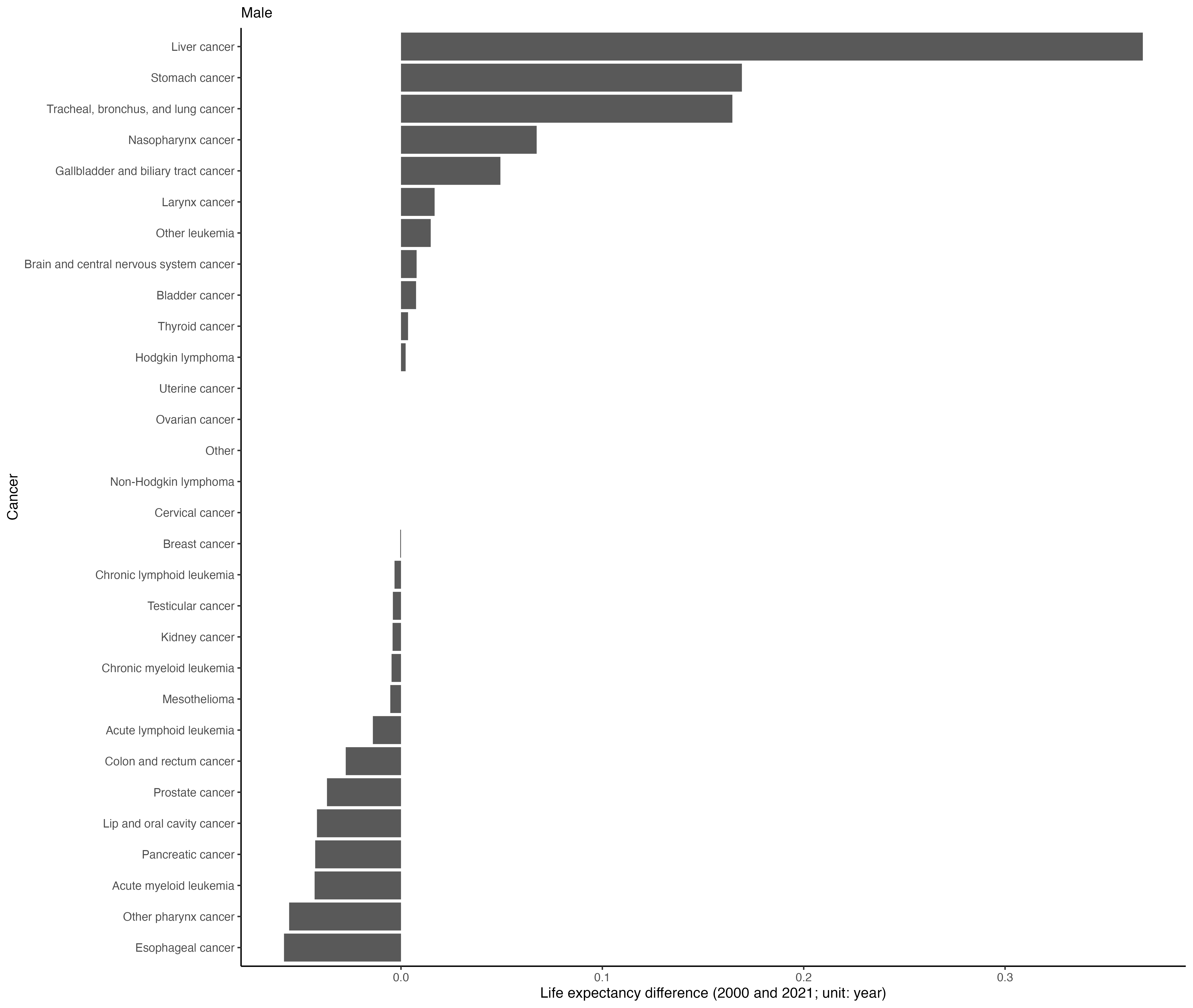


**Figure S10.** Decomposition of life expectancy contributions by cancer type in Taiwan, 2021 vs. 2000 (Male).
